# Neurological disorders caused by recreational use of nitrous oxide—a retrospective study from a German metropolitan area and review of the literature

**DOI:** 10.1186/s42466-025-00385-0

**Published:** 2025-05-04

**Authors:** Asya Tshagharyan, Se-Jong You, Christian Grefkes, Elke Hattingen, Joachim P. Steinbach, Pia S. Zeiner, Marcel Hildner, Iris Divé

**Affiliations:** 1 Department of Neurology, Goethe University Frankfurt, University Hospital, 60528 Frankfurt, Germany; 2 Institute of Neuroradiology, Goethe University Frankfurt, University Hospital, 60528 Frankfurt, Germany; 3 Dr. Senckenberg Institute of Neurooncology, Goethe University Frankfurt, University Hospital, 60528 Frankfurt, Germany

**Keywords:** Nitrous oxide, Polyneuropathy, Myelopathy, Germany

## Abstract

**Background:**

The recreational use of nitrous oxide (N_2_O) has seen a worldwide rise in the recent years, resulting in an increased incidence of neurological complications due to N_2_O-induced functional vitamin B_12_ deficiency. Here, we report on a cohort of patients admitted to a tertiary care center with neurological symptoms in the context of recreational N_2_O use between 2020 and 2024.

**Methods:**

We screened the database of the University Hospital Frankfurt for patients ≥ 18 years of age who presented with neurological deficits and a history of N_2_O consumption between January 2020 and December 2024. We analyzed the spectrum of neurological deficits as well as radiological and laboratory findings.

**Results:**

We identified a total of 20 patients, 16 males and 4 females, with a median age of 21 years. We found a steady increase in the number of cases, with no cases in 2020 and 2021 and a definite peak in 2024. The mean daily N_2_O consumption was 2500 g. All patients reported sensory deficits; 85% had gait disturbances and 70% had motor deficits. Less frequent symptoms included pain, bladder or bowel dysfunction, fatigue and spasticity. The median score on the modified Rankin scale (mRS) was 2, with some patients being wheelchair-bound. The most frequently observed lesion pattern was combined myelo-polyneuropathy. T2-hyperintense myelon lesions were observed in 11 of 15 patients (73.3%). Surprisingly, laboratory work-up revealed normal vitamin B_12_ levels in nearly all patients (95%), whereas homocysteine and methylmalonic acid levels were prominently elevated in all patients (100%). In addition, 13 patients (65%) presented with hematological abnormalities. All of the patients who presented for follow-up (20%) reported continued use of N_2_O. There was no neurological improvement in any of these cases.

**Conclusions:**

Our study confirms that the increasing incidence of N_2_O-induced neurotoxicity reported in other countries can also be observed in Germany. Therefore, it underlines the relevance of the current debate on health policies. In addition, our study highlights the pitfalls of vitamin B12 laboratory testing and emphasizes the need to address substance addiction in treatment.

## Background

Nitrous oxide (N_2_O) has been used as an anesthetic with strong analgesic and anxiolytic effects for over 150 years [12]. This substance was already used for recreational purposes in the eighteenth century, e.g. at laughing gas parties. Although the use of N_2_O has generally been considered safe, it has long been known to damage neurological function through inactivation of vitamin B_12,_ which, in turn, causes neurotoxicity by impairing myelin synthesis [15, 17, 25]. This leads to neurological symptoms, most frequently presenting as paresthesia, peripheral polyneuropathy or subacute combined degeneration of the spinal cord [10, 17]. Myelopathy primarily affects the dorsal columns and pyramidal tracts in the spinal cord. In advanced stages of myelopathy, destruction of the myelin sheaths leads to demyelination with subsequent axonal degeneration [29].

In recent years, there has been a sharp increase in the recreational use of N_2_O in the EU as well as in the USA and Canada, particularly among people aged 18–24 years [6, 8, 16, 33]. This development is accompanied by a rising incidence of neurological complications in the context of N_2_O consumption, as shown by recent studies from France and the Netherlands [4, 33].

For Germany, larger case studies are scarce. However, in the past two years, an unprecedented increase in relatively young patients with neurological complications due to vitamin B_12_ deficiency has been anecdotally reported from several large German cities [19]. Here, we performed a systematic analysis of all patients who were admitted to Frankfurt University Hospital due to N_2_O-associated neurotoxicity.

## Methods

### Patient selection

We screened the database of the University Hospital Frankfurt for patients ≥ 18 years who presented with neurological deficits and a history of recreational N_2_O consumption between January 2020 and December 2024. More specifically, we conducted a keyword search for the terms “nitrous oxide”, “laughing gas”, “vitamin B12”, “hypovitaminosis” or “subacute degeneration of the spinal cord” in both the electronic medical records as well as the radiological database of our department. Basic epidemiological data, neurological symptoms and laboratory results on vitamin B_12_ metabolism were extracted from clinical records and deidentified. MR imaging of the central nervous system was viewed whenever available.

### Data visualization and statistical analysis

For data visualization, we used GraphPad Prism 10.1.2. (Boston, MA, USA) and CorelDRAW Graphics Suite 2019 (Alludo, Ontario, Canada). Statistical analysis was performed with GraphPad Prism 10.1.2 and Microsoft Excel (Microsoft, WA, USA). Unless otherwise indicated, values are given as the means.

### Review of the literature

We searched the MEDLINE®-indexed literature using the PubMed search engine from the National Centre for Biotechnology Information (www.pubmed.gov) from January 2010 until February 2025. We used a combined search approach including the following keywords: “nitrous oxide” [MeSH terms]” OR “laughing gas” (MeSH terms) AND “myelopathy” [all fields] OR “neuropathy” [all fields]. We excluded studies with ≤ 5 patients, studies not focusing on neurological disorders and articles that were not publicly available. In addition, we examined the reference list of all identified studies and review articles for studies that might be of relevance.

## Results

### Patient cohort and nitrous oxide consumption

We identified a total of 20 patients with a history of recreational N_2_O consumption and neurological symptoms who were treated at the Department of Neurology/University Frankfurt (Table 1). The majority of patients (80%) were male. The median age of the patient cohort was 21 years (range 19–30). Patient interviews did not indicate consumption of other drugs or alcohol abuse. Most of the patients were in an apprenticeship or in a low to middle income job, none had a university degree. The reported amount of consumption of N_2_O ranged from 40 g to 8000 g per day. The mean daily consumption was 2500 g. Assuming an average quantity of 8 g per container, this corresponds to 312 balloons per day. Notably, two patients reported frequent exposure to N_2_O but consistently denied active consumption. The median duration of consumption before the first evaluation at our hospital was 11 months, with a range of 1 month to two years.

**Table 1:** Basic demographic data and consumption patterns of nitrous oxide

Sex	Age (years)	Daily consumption	Duration of consumption	Damage pattern
Male	23	2000 g	8 months	Mixed
Female	21	4000 g	Several years	Polyneuropathy
Male	19	Unknown	12 months	Mixed
Male	20	Unknown	Unknown	Myelopathy
Male	20	Unknown	5 months	Mixed
Female	20	3000–5000 g	12 months	Myelopathy
Male	21	Passive consumption	24 months	Mixed
Female	23	3000–4000 g	12 months	Mixed
Male	27	5000 g	9 months	Mixed
Male	28	160 g	18 months	Mixed
Male	23	600 g	1 month	Polyneuropathy
Male	21	40 g	Unknown	Mixed
Male	29	3000–5000 g	Unknown	Polyneuropathy
Female	20	500 g	10 months	Myelopathy
Male	19	2000 g	Unknown	Polyneuropathy
Male	19	Unknown	Unknown	Myelopathy
Male	24	1000–2000 g	12 months	Polyneuropathy
Male	25	2000–8000 g	2 months	Polyneuropathy
Male	19	Passive consumption	Unknown	Mixed
Male	23	5000 g	24 months	Myelopathy

### Increasing incidence of nitrous oxide-associated neurotoxicity between 2020 and 2024

**Fig. 1:**
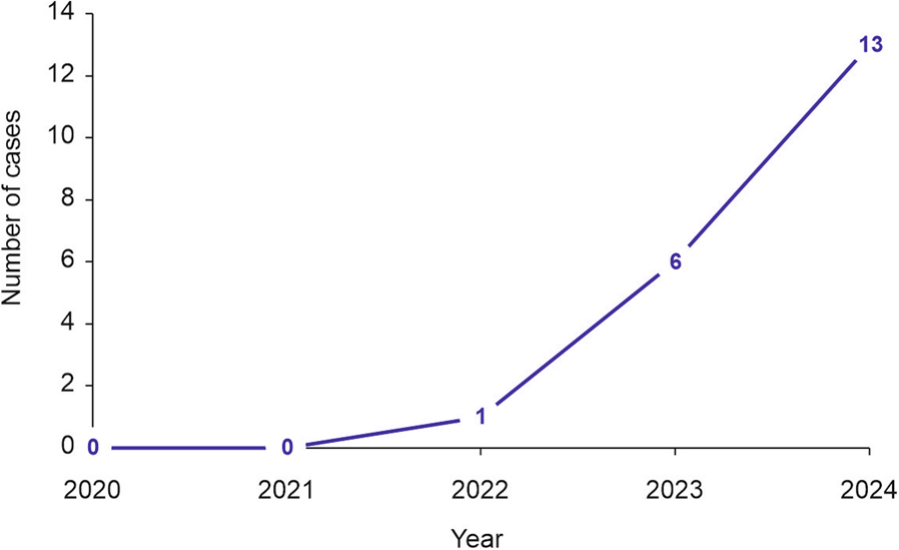
Development of the number of cases of nitrous oxide-related neurological symptoms between 2020 and 2024

During the investigated period from 2020–2024, we observed a significant increase in the number of patients treated with N_2_O-associated neurotoxicity at our department (Fig. 1). No cases occurred before 2022 according to the database of the hospital. The number of cases then rose noticeably in 2023, and reached a maximum thus far in 2024 with 13 of the 20 cases (65%).

### Clinical findings

**Fig. 2:**
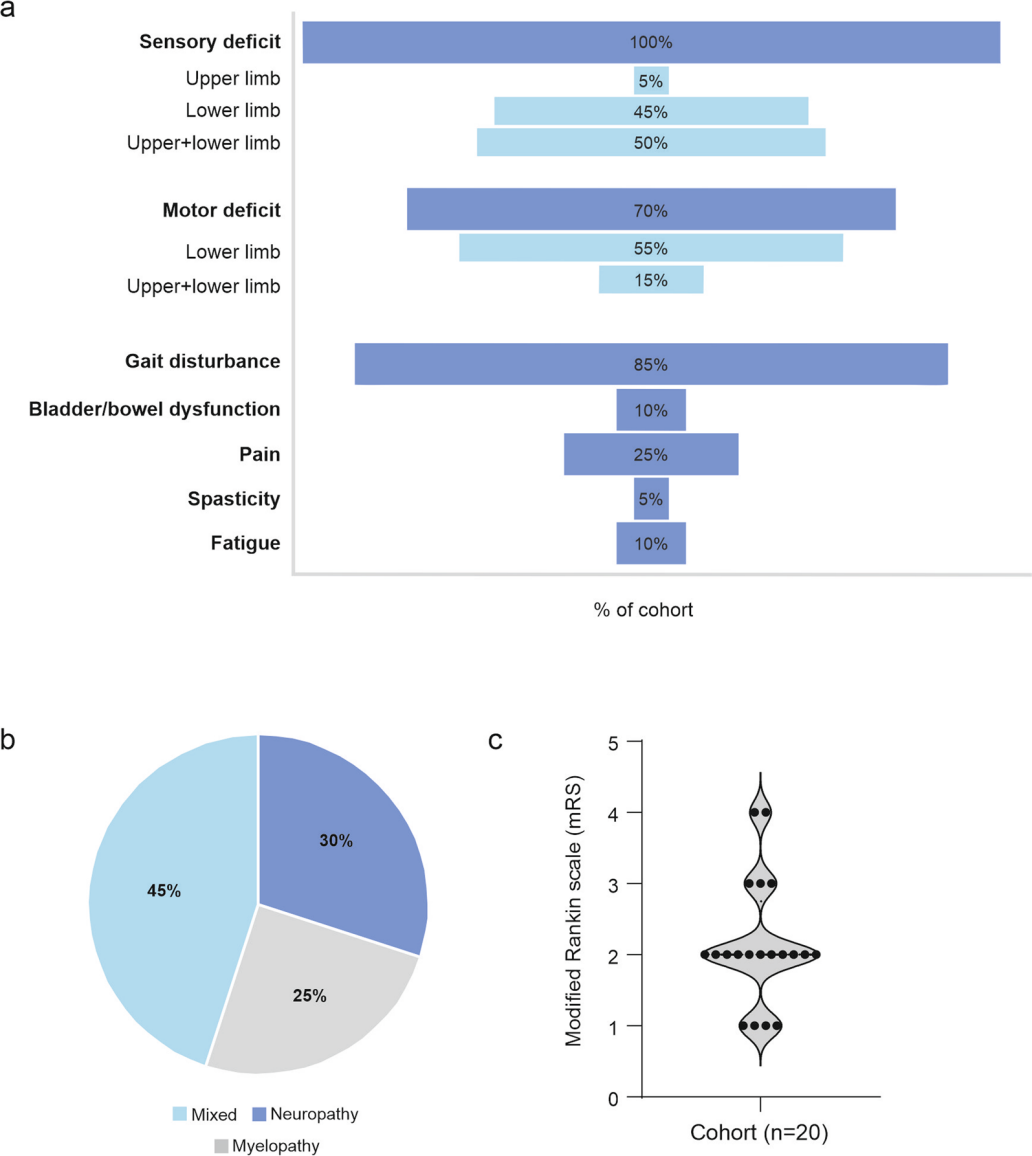
Clinical findings. **a** Frequency of neurological symptoms given as a percentage of the entire cohort. The bars in light blue are depicted as percentages of all patients reporting sensory or motor deficits. **b** Distribution of the damage pattern given as a percentage of the cohort. **c** Clinical severity quantified by the modified Rankin scale

All patients complained of sensory deficits (Fig. 2a). Most had additional neurological symptoms, presenting primarily as gait disturbance (85%, n = 17) and/or motor deficits (70%, n = 14). Pain (n = 5), bladder/bowel dysfunction (n = 2), fatigue (n = 2) and spasticity (n = 1) were reported less often. The most frequently observed clinical pattern of N_2_O-associated neurotoxicity was combined myeloneuropathy (45%), while 30% of the patients had neuropathy, and 25% had myelopathy exclusively (Fig. 2b). We observed no correlation between the quantity or duration of N_2_O consumption and the damage pattern (Table 1). The clinical severity of the symptoms at the time of the first clinical examination was 2 on the modified Rankin scale (mRS) (Fig. 2c).

### Imaging and laboratory testing

**Fig. 3:**
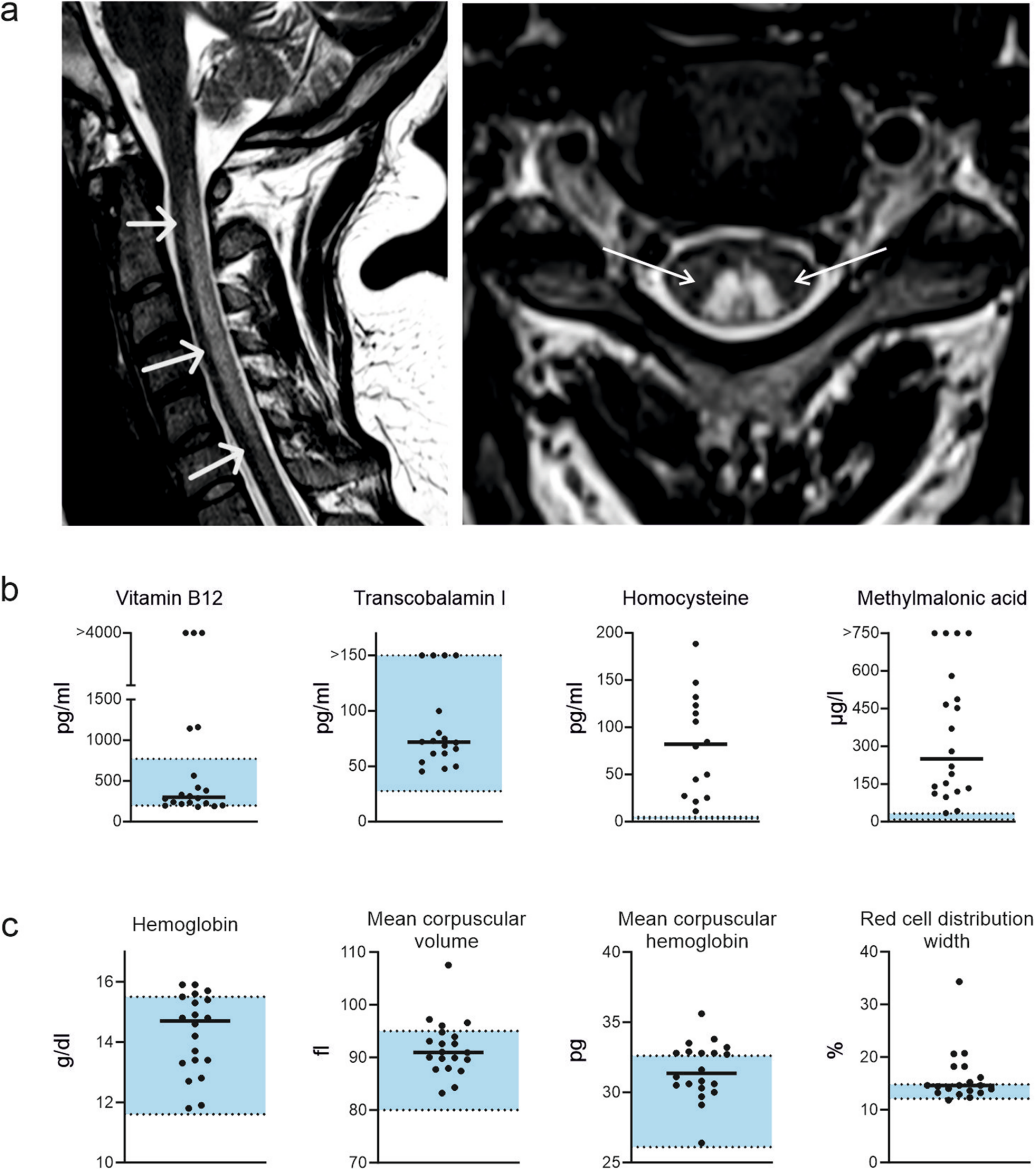
Diagnostic findings. **a** Representative T2-weighted MR image of the upper spine showing hyperintense lesions of the posterior funiculi (inverted “v” sign). **b** Results of the laboratory work-up of vitamin B_12_ metabolism. The reference ranges are marked in blue. **c** Results of the laboratory hemoglobin, mean corpuscular volume and hemoglobin and red cell distribution width. The reference ranges are marked in blue

MR imaging was performed in 15 of the 20 patients (75%). Among these patients, 11 (73.3%) had pathological hyperintensity in the T2-weighted sequences in the posterior funiculi of the spinal cord (Fig. 3a), indicating subacute combined degeneration of the spinal cord (formerly referred to as funicular myelosis). The level of vitamin B_12_ was within the normal or lower-normal range in all but one patient (Fig. 3b). Three patients even had significantly elevated vitamin B_12_ levels, which was due to self-initiated substitution prior to admission to our clinic. These patients had no particular dietary habits or other substance dependencies. All patients had normal values for transcobalamine I. In contrast, homocysteine and methylmalonic acid were well above the upper limit of normal in all 20 patients. Thirteen out of 20 patients (65%) had at least one hematological abnormality. These included alterations in the mean corpuscular hemoglobin (MCH), volume (MCV) or red cell distribution width (RCDW) (Fig. 3c). No patient had low hemoglobin levels.

### Treatment and follow-up

All patients were treated with 1000 µg of vitamin B_12_ per day i.m. for 10 days followed by 1000 μg of vitamin B_12_ weekly or monthly. We recommend determining the dosage and duration of treatment on the basis of the level of methylmalonic acid at follow-up. Of note, only 4 of the 20 patients (20%) presented for a neurological follow-up examination in our department within three months after discharge. None of these four patients experienced any improvement in their clinical condition. Notably, all four patients continued to use N_2_O.

## Discussion

### The rising popularity of recreational nitrous oxide use

Between 2020 and 2024, we observed an unprecedented increase in relatively young adults who presented with N_2_O-induced neurological symptoms. This coincides with an increased consumption of N_2_O among adolescents in the catchment area of our hospital as evidenced by a recent urban drug report [21, 22]. In this report, the lifetime prevalence of N_2_O increased from 7% in 2020 to 17% in 2022 [22] and 14% in 2023 [23]. More than 60% of the survey participants reported high popularity of this substance among their peers. The increase in the recreational use of N_2_O has been reported by other European countries and worldwide [5, 6].

In our cohort, 80% were male and had a median age of 21 years. This finding is in line with the majority of studies published on recreational N_2_O use between 2010 and 2025 (Table 2). Specifically, 17 out of 24 studies had a majority of male patients and the median age across all studies was 22 years. Studies from France and the Netherlands have identified a low level of education, employment in the low-wage sector or unemployment as factors that predict a high prevalence of laughing gas consumption [4, 32]. In addition, surveys from the Netherlands have found that consumers often had a non-Dutch background. It must be emphasized that socioeconomic data was rarely collected in the studies we identified. Hence, representative surveys at national level are needed to better understand the socioeconomic and cultural background of the consumers.

**Table 2:** Summary of studies on N_2_O-induced neurotoxicity between 2010 and 2025

	Gao et al Front Neurol 2023	Meißner et al NRP 2025	Fang et al J Clin Neurol 2023	Keddie et al J Neurol 2018	Bao et al Neuropsychiatr Dis Treat 2020	Tuan et al Neurol Int 2020	Zheng et al., Neuropsychiatr Dis Treat 2020	Zhang et al., Front Neurol 2021	Vollhardt et al., J Neurol 2021
Year of recruitment	2016–2021	2020–2024	2018–2020	2016–2017	2015–2019	2018–2019	2016–2019	2018–2020	2020–2021
Country	China	Germany	China	England	China	Vietnam	China	China	France
Number of patients	20	23	76	10	33	47	21	20	12
Age (mean, range)	22 (16–30)	25	21 (14–33)	22 (median) (17–26)	22 (14–27)	24 (15–50)	23 (15–34)	23 (median)(20–28)	22 (17–28)
*Sex*									
Male	25%	65%	45%	70%	88%	49%	67%	55%	50%
Female	75%	35%	55%	30%	12%	51%	33%	45%	50%
Exposure time to N_2_O	7 months	NR	12 months	NR	18 ± 4.3 months	8.8 months	7.2 months	NR	12.7 months
Pattern of N_2_O use	NR	83% regular consumption (15/18)	NR	2–3 days/week (average); 72–2000 canisters/week	NR	3.2 days/week, 36 balloons/day	NR	NR	3,109 ± 2,800 g/week (mean)
*Clinical syndrom*									
Sensory deficit	70%	96%	98%	100%	91%	100%	86%	95%	92%
Motor deficit	95%	52%	79%	50%	82%	87%	90%	80%	75%
Gait disturbance	95%	83%	42%	80%	46%	–	–	20%	–
Bladder/bowel	5%	–	17%	10%	6%	–	–	5%	50%
Decreased reflexes	100%	74%	59%	50%	85%	85%	67%	–	83%
Increased reflexes	–	–	–	30%	15%	–	10%	–	–
Spasticity/UMN*	–	13%	13%	30%	–	–	–	–	8%
*Damage pattern*									
Myelopathy	NA	60% (14/21)	56% (20/36)	100% (9/9)	48%	68%	47% (8/17)	79% (15/19)	75%
Neuropathy	100% (15/15)	87%	NR	NR	100%	NR	85% (17/20)	89% (16/18)	83%
*Hematology*									
Low Hb	40% (4/10)	26% (6/23)	86% (44/51)	NR	6%	32%	10% (2/20)	15%	NR
Increased MCV	20% (2/10)	NR	34% (15/44)	0%	6%	NR	15% (3/20)	25%	NR
*Vitamin B12 deficiency*	57% (4/7)	35% (8/23)	64% (28/44)	40%	27%	57%	17% (3/18)	39% (5/13)	42%
Increased Hcy	60% (3/5)	89% (8/9)	71% (25/35)	NR	82%	87%	78% (14/18)	88% (14/16)	100% (11/11)
Increased MMA	NR	95% (18/19)	NR	88% (7/8)**	NR	NR	NR	NR	100% (11/11)
*Outcome*									
Improvement at discharge	75% (15/20)	NR	NR	NR	NR	NR	100%	80%	NR
Improvement at follow-up	NR		NR		NR	NR			
Complete		–		30% (2/6)			80% (4/5)	94% (16/17)	–
Partial		43% (3/7)		50% (3/6)			20% (1/5)	6% (1/17)	100% (8/8)
None		57% (4/7)		20% (1/6)			–	–	–

	Nugteren-Van Lonkhuyzen et al. [24]	Cruz et al Eur J Neurol.2024	Soderstrom et al Druc Alcohol Rev 2024	Dabby et al Isr Med Assoc J. 2024	Van Riel et al Int J Drug Policy 2022	B J Rujiter et al J Neurol.2024	Hassing et al Eur J Neurol. 2024	Dawudi et al J Neurol 2024	Fortanier et al Eu J Neurol 2023
Year of recruitment	2021–2022	2020–2024	2019–2021	2022–2023	2010–2020	2016–2021	2018–2021	2018–2021	2020–2023
Country	The Netherlands	France	Australia	Israel	The Netherlands	The Netherlands	The Netherlands	France	France
Number of patients	101	61	22	8	433	251	70	181	58
Age (mean, range)	23 (16–26) (median)	24	22 (median) (20–50)	21 (median) (17–32)	22 (median)	22 (IQR 20–26)	22 (median) (18–50)	23	23
*Sex*									
Male	52%	62%	68%	62%	64%	63%	64%	54%	67%
Female	48%	38%	32%	38%	36%	37%	36%	46%	33%
Exposure time to N_2_O	NR	9 months	9 months	NR	NR	11 months	12 months	6 months	8.7 ± 5.2 months
Consumption pattern	77% heavy use (> 50 balloons in one session or use from a tank)	4–19 600-g-cylinders/week (median)	150 N_2_O bulbs/day (median)	NR	59% heavy use (> 50 balloons in one session or use from a tank)	1.6 kg per day (0.5–4.0 kg)	4 kg/week	1200 g/day (average)	10.8 ± 11.6 L/week
*Clinical syndrom*		NR			NR	NR			
Sensory deficit	41%		63%	63%			97%	98%	100%
Motor deficit	13%		22%	25%			57%	72%	60%
Gait disturbance	9%		59%	75%			77%	84%	88%
Bladder/bowel	–		14%	–			20%/10%	19%	28%
Decreased reflexes	–		–	13%			67%	–	74%
Increased reflexes	–		–	25%			9%	–	–
Spasticity/UMN*	–		–	–			9%	–	3%–
*Damage pattern*									
Myelopathy	NR	79% (42/53)	55%	63%	NR	41%	55%	63%	54% (26/48)
Neuropathy	41%	66% (35/53)	NR	38%	NR	78%	81%	75%	NR
*Hematology*	NR	NR	NR	NR	NR	NR	NR	NR	
Low Hb									9% (5/57)
Increased MCV									
*Vitamin B12 deficiency*	NR	NR	55%	75%	NR	40%	50% (34/67)	NR	51%
Increased Hcy			93% (15/16)	85% (6/7)		92% (109/118)	–		96%
Increased MMA			NR	100% (5/5)		NA	–		NR
*Outcome*									
Improvement at discharge	NR	NR	NR	100%	NR	NR	NR	NR	NR
Improvement at follow-up		NR	NR	NR	NR	79% partial or complete recovery (80/102)			NR
Complete	86% (6/7)						31% (16/52)	8% (5/64)	
Partial	14% (1/7)						50% (26/52)		
None							19% (10/52)		

	Qin et al J peripher Nerv Syst, 2022	Largeau et al Eur J Neurol 2022	Li et al Brain Behav 2021	Jiang et al Brain behav, 2021	Swart et al Eur J Neurol 2021	Yu et al Brain Behav. 2022
Year of recruitment	2019 -2020	2019–2020	2017–2020	2017–2020	2016–2020	2018–2020
Country	China	France	China	China	Australia	China
Number of patients	15	20	61	63	20	110
Age (mean, range)	22 (19–33)	19 (median) (16–34)	22	23 (15–33)	24 (18–40)	21 (14–33)
*Sex*						
Male	47%	85%	69%	60%	45%	52%
Female	53%	15%	31%	40%	55%	48%
Exposure time to N_2_O	2.5 months	6 months	8.5 ± 7.7 months	NR	9 months	12.5 ± 4.2 months
Consumption pattern	26 canisters/month (median)	100 cartridges/day; 58% showed daily exposure	NR	4000 (2400 − 7000) ml per session, intake frequency 3.33 ± 1.69 times per week	148 canisters/day (averaged)	NR
*Clinical syndrom*						
Sensory deficit	100%	100%	80%	100%	100%	80%
Motor deficit	100%	25%	57%	43%-	45%	83%
Gait disturbance	53%	100%	66%	97%	100%	–
Bladder/bowel dysfunction	–	–	8%	-	20%	10%
Decreased reflexes	60%	–	49%	47%	15%	71%
Increased reflexes	20%	–	10%	–	15%	9%
Spasticity/UMN*	53%	–	23%	36%	15%	7%
*Damage pattern*	NR					
Myelopathy		64% (7/11)	49%	60%	100% (20/20)	52% (25/50)
Neuropathy		36% (4/11)	80%	81%	100% (6/6)	100% (87/87)
*Hematology*						
Low Hb	NR	NR	5%	20%	45%	35% (25/71)
Increased MCV			NR	22%	NR	20% (5/25)
*Vitamin B12 deficiency*	33% (3/9)	64% (9/14)	44% (20/45)	35%	50%	60% (34/57)
Increased Hcy	NR	100% (13/13)	68% (27/40)	87%	83% (10/12)	69% (31/45)
Increased MMA	NR	100% (7/7)	NR	NR	NR	NR
*Outcome*						
Improvement at discharge	NR	NR	NR	NR	NR	NR
Improvement at follow-up		NR		NR		
Complete	87%		95% (58/61)		13% (1/8)	67% (34/51)
Partial	13%		5% (3/61)		87% (7/8)	33% (17/51)
None	–		–		–	2% (1/51)

UMN = upper motor neuron; NR = not reported; NA = not publicly available; Hcy = homocysteine; MMA = methylmalonic acid; MCV = mean corpuscular volume

*Including positive Babinski sign

**Assessed only in patients with normal vitamin B12 levels

The rising popularity of N_2_O worldwide has been attributed in part to its perception as a harmless substance [21, 23] as well as to its easy-access supply, e.g., through online shops and kiosks [6, 21]. A recent development is the sale of large cylinders of up to 2 kg, allowing for heavy use at reduced costs. In a recent study from France involving 181 patients between 2019 and 2021, the average daily consumption was 1200 g [4]. Notably, the authors reported that this number was 27,000 times greater than that reported in 2017 for the same urban area. In our cohort, 85% of the patients with active use consumed ≥ 400 g/day, and the mean daily consumption was 2500 g. Hence, the growing number of patients presenting with neurological sequelae from N_2_O use could also result from a subgroup of users with excessive daily intake. Nevertheless, cases of neurological damage resulting from infrequent or passive consumption have also been reported [28, 30], which was also observed in our study. Consequently, no safe level of use can be defined at this point.

In our literature search, it was striking that nitrous oxide consumption was either not reported at all or that consumption was quantified in very heterogeneous units, which limited comparability. Some of the authors reported that patients did not want to provide any information on their consumption or that the information given was very vague. Clinicians should take this into account when taking the patient’s medical history.

### Diagnostic findings

A crucial aspect for clinical management is the diagnosis at the earliest timepoint. From a clinical point of view, sensory deficits (hyp-/paresthesia) are the most common, often accompanied by gait disturbance, muscle weakness and reduced muscle reflexes (Table 2, Fig. 2). It is therefore not surprising that subacute polyneuropathy is often assumed as the initial differential diagnosis [9]. Myelopathy was present in about every second patient in our literature search. However, symptoms that suggest damage of the CNS (e.g. spasticity, increased tendon reflexes or pathologic reflexes) were often not detectable.

It has been reported that the neurotoxicity of N_2_O is dose dependent [34]. Furthermore, the patterns of use could impact the clinical symptoms of patients in that the effect on the spinal cord is dependent on the quantity of N_2_O, whereas the presence of polyneuropathy may depend on the duration of exposure to N_2_O [3]. We did not observe such a correlation in our cohort, although this could be due to the limited number of cases.

In the case of suspected or confirmed nitrous oxide consumption, the diagnostic work-up primarily includes laboratory evaluation for vitamin B_12_ metabolism. However, the use of vitamin B_12_ levels for diagnosis remains problematic because of its low specificity. Although studies have shown reduced vitamin B_12_ levels in up to 70% of consumers [26, 35], our literature search underlines that total circulating vitamin B_12_ is not a reliable indicator of nitrous oxide-induced neurological disorders. Low vitamin B_12_ levels varied between 17% and 75%. In our cohort, only one patient (5%) had low vitamin B_12_ levels. In addition, it must be emphasized that in some studies, including ours, patients had already started self-treatment with vitamin B_12_ before seeking medical help. Thus, relying on vitamin B_12_ levels can mask the functional impairment of vitamin B_12_ metabolism.

The inactivation of vitamin B_12_ by N_2_O incapacitates the enzymatic activity of methylmalonyl-CoA-mutase and methionine synthase, resulting in the accumulation of both methylmalonic acid and homocysteine [2]. In all studies in which both vitamin B_12_ and homocysteine were examined, homocysteine was significantly more frequently elevated. This is in line with our own findings, as well as a recent review [20]. Hence, homocysteine and methylmalonic acid are much more reliable markers for N_2_O-induced disruption of vitamin B_12_ metabolism and for monitoring treatment success [11, 20]. Since the analysis of methylmalonic acid is costly, homocysteine is usually preferred. This is reflected in the results of our literature search: methylmalonic acid was only examined in 5 of 24 studies. Whether homocysteine or methylmalonic acid are indicative of the mechanisms of N_2_O-induced neurotoxicity is still under investigation [13].

### Treatment and outcome

The cornerstone of treatment is vitamin B_12_ supplementation. There is a consensus that supplementation should initially be carried out at high intensity, although there are different schemes for implementation. It is recommended that the substitution is initially carried out daily [14] or every other day [27] via i.m. injections of 1000 µg of hydroxycobalamin over the course of one to two weeks or until there is no further improvement of symptoms. Supplementation can then be reduced to a weekly or 3-monthly dose, depending on whether the patient had a pre-existing vitamin B_12_ deficiency.

There are little data on long-term follow-up, which is reflected in our literature search. In almost all studies that reported on the clinical outcome, including ours, a high rate of loss to follow-up was observed. Furthermore, information on N_2_O abstinence was frequently missing.

Overall, a high percentage of patients show some neurological improvement (Table 2), either at discharge or at follow-up. In the 13 studies with follow-up, the percentage of complete recovery ranged from 0 to 95%. In a Chinese review with 51 patients at follow-up, 67% recovered completely whereas 33% had residual symptoms [35]. Notably, this study recorded only one relapse during the observation period. In two systematic case reviews, partial recovery was most frequently observed [10, 18], and progression of the disease was only seen if patients continued consumption. This could explain the poor outcome of our follow-up patients and emphasizes that cessation of N_2_O consumption is crucial for recovery [18].

Reports of excessive recreational use have foregrounded the debate of whether N_2_O has addictive potential. Previous studies have shown that the majority of heavy users show symptoms of substance use disorders, with up to 90% reporting the use of N_2_O in larger quantities and for longer periods than intended [1, 7, 24]. In addition to psychological addiction, reports have shown that frequent users tend to inhale larger quantities over time to experience similar physical effects [31]. In our cohort, all patients who presented for follow-up continued to use N_2_O despite their considerable neurological deficits. In a Dutch study, 80% of the participants unsuccessfully tried to cease N_2_O use [24]. This highlights the need to address substance addiction in treatment.

## Conclusions

In summary, the increasing number of patients presenting with neurological sequelae from the use of N_2_O could be a result of several factors, including its rising popularity, low costs, excessive intake fueled by widespread availability, and the addictive potential of the gas. These aspects explain why some countries, e.g. France and the Netherlands, have introduced health policy changes that strongly restrict open access to N_2_O [5]. In Germany, changes in legislation have not yet been implemented. Our study from a tertiary care center in Germany confirms the increasing incidence of N_2_O-induced neurotoxicity especially in young adults that has been observed in other countries. It therefore underlines the relevance of the current health policy debate. Target group-specific measures to raise awareness of the health risks appear necessary, for example via social media or schools. The high median daily consumption in our cohort and the continued use observed at follow-up stress the addictive potential of N_2_O. Addiction counseling should therefore be an integral part of treatment.

## Data Availability

All data generated or analyzed during this study are included in this published article.

## Abbreviations

MCH: Mean corpuscular hemoglobin
MCV: Mean corpuscular volume
mRS: Modified Rankin scale
N_2_O: Nitrous oxide
RCDW: Red cell distribution width

## References

[1] Back, S., Kroon, E., Colyer-Patel, K., & Cousijn, J. (2024). Does nitrous oxide addiction exist? An evaluation of the evidence for the presence and prevalence of substance use disorder symptoms in recreational nitrous oxide users. *Addiction (Abingdon, England),**119*(4), 609–618. 10.1111/add.16380 37904333 10.1111/add.16380

[2] Chanarin, I. (1982). The effects of nitrous oxide on cobalamins, folates, and on related events. *Critical Reviews in Toxicology,**10*(3), 179–213. 10.3109/10408448209037455 6127188 10.3109/10408448209037455

[3] Cruz, E. S., Fortanier, E., Hilezian, F., Maarouf, A., Boutière, C., Demortière, S., Rico, A., Delmont, E., Pelletier, J., Attarian, S., & Audoin, B. (2024). Factors affecting the topography of nitrous oxide-induced neurological complications. *European Journal of Neurology,**31*(7), e16291. 10.1111/ene.16291 38532638 10.1111/ene.16291 PMC11235663

[4] Dawudi, Y., Azoyan, L., Broucker, T. D. E., Gendre, T., Miloudi, A., Echaniz-Laguna, A., Mazoyer, J., Zanin, A., Kubis, N., Dubessy, A.-L., Gorza, L., Ben Nasr, H., Caré, W., d’Izarny-Gargas, T., Formoso, A., Vilcu, A.-M., & Bonnan, M. (2024). Marked increase in severe neurological disorders after nitrous oxide abuse: A retrospective study in the Greater Paris area. *Journal of Neurology,**271*(6), 3340–3346. 10.1007/s00415-024-12264-w 38478030 10.1007/s00415-024-12264-w PMC11136741

[5] European Monitoring Centre for Drugs and Drug Addiction. *European Drug Report 2024: Trends and Developments*. https://www.euda.europa.eu/publications/european-drug-report/2024_en

[6] European Monitoring Centre for Drugs and Drug Addiction 2022. *Recreational use of nitrous oxide: a growing concern for Europe*. https://www.euda.europa.eu/publications/rapid-communication/recreational-use-nitrous-oxide-growing-concern-europe_en

[7] Fidalgo, M., Prud’homme, T., Allio, A., Bronnec, M., Bulteau, S., Jolliet, P., & Victorri-Vigneau, C. (2019). Nitrous oxide: What do we know about its use disorder potential? Results of the french monitoring centre for addiction network survey and literature review. *Substance Abuse,**40*(1), 33–42. 10.1080/08897077.2019.1573210 30913001 10.1080/08897077.2019.1573210

[8] Forrester, M. B. (2021). Nitrous oxide misuse reported to two United States data systems during 2000–2019. *Journal of Addictive Diseases,**39*(1), 46–53. 10.1080/10550887.2020.1813361 32875958 10.1080/10550887.2020.1813361

[9] Fortanier, E., Berling, E., Zanin, A., Le Guillou, A., Micaleff, J., Nicolas, G., Lozeron, P., & Attarian, S. (2023). How to distinguish Guillain-Barré syndrome from nitrous oxide-induced neuropathy: A 2-year, multicentric, retrospective study. *European Journal of Neurology,**30*(10), 3296–3306. 10.1111/ene.15998 37494104 10.1111/ene.15998

[10] Garakani, A., Jaffe, R. J., Savla, D., Welch, A. K., Protin, C. A., Bryson, E. O., & McDowell, D. M. (2016). Neurologic, psychiatric, and other medical manifestations of nitrous oxide abuse: A systematic review of the case literature. *The American Journal on Addictions,**25*(5), 358–369. 10.1111/ajad.12372 27037733 10.1111/ajad.12372

[11] Gernez, E., Lucas, A., Niguet, J.-P., Bennis, A., Diesnis, R., Noyce, A. J., & Grzych, G. (2024). What biological markers could be used for diagnosis and monitoring of nitrous oxide abuse? *European Journal of Neurology,**31*(3), e16188. 10.1111/ene.16188 38117540 10.1111/ene.16188 PMC11235905

[12] Gillman, M. A. (2019). Mini-review: A brief history of nitrous oxide (n2o) use in neuropsychiatry. *Current Drug Research Reviews,**11*(1), 12–20. 10.2174/1874473711666181008163107 30829177 10.2174/1874473711666181008163107 PMC6637098

[13] Grzych, G., Scuccimarra, M., Plasse, L., Gernez, E., Cassim, F., Touze, B., Girot, M., Bossaert, C., & Tard, C. (2024). Understanding neuropathy features in the context of nitrous oxide abuse: A combined electrophysiological and metabolic approach. *Biomedicines*. 10.3390/biomedicines12020429 38398031 10.3390/biomedicines12020429 PMC10886673

[14] de Halleux, C., & Juurlink, D. N. (2023). Diagnosis and management of toxicity associated with the recreational use of nitrous oxide. *CMAJ: Canadian Medical Association Journal= Journal De L’association Medicale Canadienne,**195*(32), E1075–E1081. 10.1503/cmaj.230196 37604519 10.1503/cmaj.230196 PMC10442242

[15] Hathout, L., & El-Saden, S. (2011). Nitrous oxide-induced B₁₂ deficiency myelopathy: Perspectives on the clinical biochemistry of vitamin B₁₂. *Journal of the Neurological Sciences,**301*(1–2), 1–8. 10.1016/j.jns.2010.10.033 21112598 10.1016/j.jns.2010.10.033

[16] Kaar, S. J., Ferris, J., Waldron, J., Devaney, M., Ramsey, J., & Winstock, A. R. (2016). Up: The rise of nitrous oxide abuse. An international survey of contemporary nitrous oxide use. *Journal of Psychopharmacology (Oxford England),**30*(4), 395–401. 10.1177/0269881116632375 26912510 10.1177/0269881116632375

[17] Layzer, R. B. (1978). Myeloneuropathy after prolonged exposure to nitrous oxide. *Lancet (London, England),**2*(8102), 1227–1230. 10.1016/S0140-6736(78)92101-3 82736 10.1016/s0140-6736(78)92101-3

[18] Marsden, P., Sharma, A. A., & Rotella, J.-A. (2022). Review article: Clinical manifestations and outcomes of chronic nitrous oxide misuse: A systematic review. *Emergency Medicine Australasia : EMA,**34*(4), 492–503. 10.1111/1742-6723.13997 35695047 10.1111/1742-6723.13997

[19] Meißner, J. N., Neuneier, J., Bartzokis, I., Rehm, M., Al-Hayali, A., Müller, M., Paus, S., Limmroth, V., Fink, G. R., Petzold, G. C., & Nitsch, L. (2025). Increase of nitrous oxide-induced neurological disorders - a German multicenter experience. *Neurological Research and Practice,**7*(1), 3. 10.1186/s42466-024-00361-0 39815374 10.1186/s42466-024-00361-0 PMC11737043

[20] Ménétrier, T., & Denimal, D. (2023). Vitamin B12 status in recreational users of nitrous oxide: A systematic review focusing on the prevalence of laboratory abnormalities. *Antioxidants (Basel, Switzerland)*. 10.3390/antiox12061191 37371921 10.3390/antiox12061191 PMC10294871

[21] MoSyD 2022. *Monitoring system drogentrends*. https://www.uni-frankfurt.de/146663665/MoSyD_Jahresbericht_2022_final.pdf

[22] MoSyD 2023. *Monitoring system drogentrends*. https://www.frankfurt-university.de/fileadmin/standard/ISFF/MoSyD-Jahresbericht_2023_final.pdf

[23] Nabben, T., Weijs, J., & van Amsterdam, J. (2021). Problematic Use of Nitrous Oxide by Young Moroccan-Dutch Adults. *International Journal of Environmental Research and Public Health*. 10.3390/ijerph18115574 34071087 10.3390/ijerph18115574 PMC8197142

[24] Nugteren-Van Lonkhuyzen, J. J., van der Ben, L., van den Hengel-Koot, I., & S, Lange de, DW., Riel van, A. J. H. P., & Hondebrink, L,. (2023). High Incidence of Signs of Neuropathy and Self-Reported Substance Use Disorder for Nitrous Oxide in Patients Intoxicated with Nitrous Oxide. *European Addiction Research,**29*(3), 202–212. 10.1159/000530123 37100039 10.1159/000530123 PMC10389788

[25] Nunn, J. F. (1984). Interaction of nitrous oxide and vitamin B12. *Trends in Pharmacological Sciences,**5*, 225–227. 10.1016/0165-6147(84)90426-7

[26] Oussalah, A., Julien, M., Levy, J., Hajjar, O., Franczak, C., Stephan, C., Laugel, E., Wandzel, M., Filhine-Tresarrieu, P., Green, R., & Guéant, J.-L. (2019). Global burden related to nitrous oxide exposure in medical and recreational settings: A systematic review and individual patient data meta-analysis. *Journal of Clinical Medicine*. 10.3390/jcm8040551 31018613 10.3390/jcm8040551 PMC6518054

[27] Paris, A., Lake, L., Joseph, A., Workman, A., Walton, J., Hayton, T., Evangelou, N., Lilleker, J. B., Ayling, R. M., Nicholl, D., & Noyce, A. J. (2023). Nitrous oxide-induced subacute combined degeneration of the cord: Diagnosis and treatment. *Practical Neurology,**23*(3), 222–228. 10.1136/pn-2022-003631 36813556 10.1136/pn-2022-003631 PMC10313972

[28] Patel, K. K., Mejia Munne, J. C., Gunness, V. R. N., Hersey, D., Alshafai, N., Sciubba, D., Nasser, R., Gimbel, D., Cheng, J., & Nouri, A. (2018). Subacute combined degeneration of the spinal cord following nitrous oxide anesthesia: A systematic review of cases. *Clinical Neurology and Neurosurgery,**173*, 163–168. 10.1016/j.clineuro.2018.08.016 30144777 10.1016/j.clineuro.2018.08.016

[29] Renard, D., Dutray, A., Remy, A., Castelnovo, G., & Labauge, P. (2009). Subacute combined degeneration of the spinal cord caused by nitrous oxide anaesthesia. *Neurological Sciences,**30*(1), 75–76. 10.1007/s10072-009-0013-2 19169627 10.1007/s10072-009-0013-2

[30] Sluyts, Y., Pals, P., Amir, R., & Vanherpe, P. (2021). Recreational use of nitrous oxide may cause collateral neurological damage. *Acta Neurologica Belgica,**121*(4), 1097–1099. 10.1007/s13760-021-01740-z 34213739 10.1007/s13760-021-01740-z

[31] van Amsterdam, J., Nabben, T., & van den Brink, W. (2015). Recreational nitrous oxide use: Prevalence and risks. *Regulatory Toxicology and Pharmacology : RTP,**73*(3), 790–796. 10.1016/j.yrtph.2015.10.017 26496821 10.1016/j.yrtph.2015.10.017

[32] van den Toren, S. J., van Grieken, A., & Raat, H. (2021). Associations of socio-demographic characteristics, well-being, school absenteeism, and substance use with recreational nitrous oxide use among adolescents: A cross-sectional study. *PLoS ONE,**16*(2), e0247230. 10.1371/journal.pone.0247230 33600449 10.1371/journal.pone.0247230 PMC7891713

[33] van Riel, A. J. H. P., Hunault, C. C., van den Hengel-Koot, I. S., Nugteren-van Lonkhuyzen, J. J., de Lange, D. W., & Hondebrink, L. (2022). Alarming increase in poisonings from recreational nitrous oxide use after a change in EU-legislation, inquiries to the Dutch Poisons Information Center. *The International Journal on Drug Policy,**100*, 103519. 10.1016/j.drugpo.2021.103519 34753046 10.1016/j.drugpo.2021.103519

[34] Winstock, A. R., & Ferris, J. A. (2020). Nitrous oxide causes peripheral neuropathy in a dose dependent manner among recreational users. *Journal of Psychopharmacology (Oxford, England),**34*(2), 229–236. 10.1177/0269881119882532 31679459 10.1177/0269881119882532

[35] Yu, M., Qiao, Y., Li, W., Fang, X., Gao, H., Zheng, D., & Ma, Y. (2022). Analysis of clinical characteristics and prognostic factors in 110 patients with nitrous oxide abuse. *Brain and Behavior,**12*(4), e2533. 10.1002/brb3.2533 35307992 10.1002/brb3.2533 PMC9015005

